# miRNA-mediated control of exogenous *OCT4* during mesenchymal-epithelial transition increases measles vector reprogramming efficiency

**DOI:** 10.1016/j.omtm.2021.11.012

**Published:** 2021-11-29

**Authors:** Ramya Rallabandi, Brenna Sharp, Conrad Cruz, Qi Wang, Alexis Locsin, Christopher B. Driscoll, Ella Lee, Tim Nelson, Patricia Devaux

**Affiliations:** 1 Department of Molecular Medicine, Mayo Clinic, Rochester, MN 55905, USA; 2 Virology and Gene Therapy Graduate Track, Mayo Clinic Graduate School of Biomedical Sciences, Mayo Clinic, Rochester, MN 55905, USA; 3 Regenerative Sciences PhD Program, Mayo Clinic Graduate School of Biomedical Sciences, Mayo Clinic, Rochester, MN 55905, USA; 4 Department of Molecular Pharmacology and Experimental Therapeutics, Mayo Clinic College of Medicine, Rochester MN 55905, USA

**Keywords:** measles virus, reprogramming, iPSC, microRNA targeting, OCT4, mesenchymal-epithelial transition, viral vectors

## Abstract

*OCT4* is a key mediator of induced pluripotent stem cell (iPSC) reprogramming, but the mechanistic insights into the role of exogenous *OCT4* and timelines that initiate pluripotency remain to be resolved. Here, using measles reprogramming vectors, we present microRNA (miRNA) targeting of exogenous *OCT4* to shut down its expression during the mesenchymal to the epithelial transition phase of reprogramming. We showed that exogenous *OCT4* is required only for the initiation of reprogramming and is dispensable for the maturation stage. However, the continuous expression of *SOX2*, *KLF4*, and *c-MYC* is necessary for the maturation stage of the iPSC. Additionally, we demonstrate a novel application of miRNA targeting in a viral vector to contextually control the vector/transgene, ultimately leading to an improved reprogramming efficiency. This novel approach could be applied to other systems for improving the efficiency of vector-induced processes.

## Graphical abstract

**Figure:**
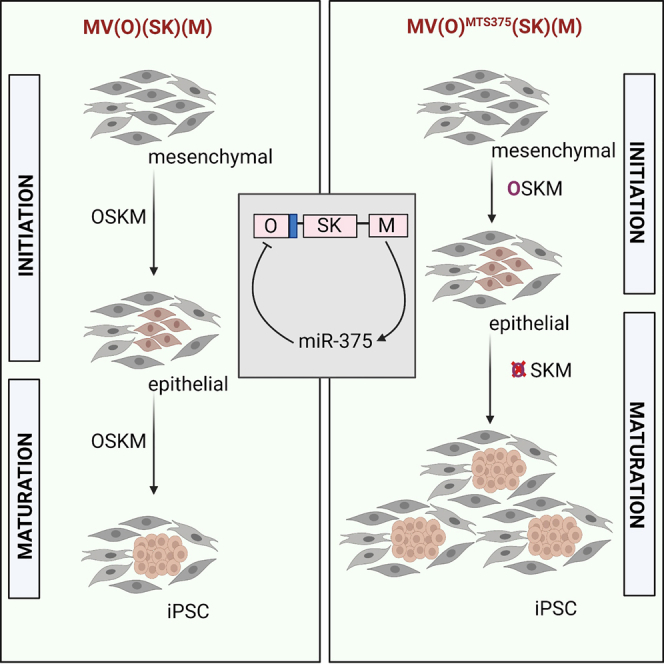

## Teaser

Devaux and colleagues develop a novel miRNA-targeted measles vector that contextually suppress exogenous OCT4 during mesenchymal to epithelial transition (MET) and improve human reprogramming efficiency and kinetics. They show that while SOX2, KLF4, and cMYC are required for both the initiation and maturation stage of reprogramming, OCT4 is essential only for the initiation stage.

## Introduction

Reprogramming is a multi-dynamic molecular process involving conversion of somatic cells to induced pluripotent stem cells (iPSCs) via overexpression of four reprogramming factors (RFs): *Oct4, Sox2, Klf4,* and *c-Myc* (OSKM)), which can be delivered using multiple vector systems.^1^, ^2^, ^3^ Based on gene expression profiling, human somatic cell reprogramming is divided into two stages; initiation and maturation, linked by mesenchymal to epithelial transition (MET) phase, which is characterized by the activation of epithelial genes and repression of mesenchymal genes.^4^, ^5^, ^6^ MET is orchestrated by exogenous RFs-OSKM.^4^, ^5^, ^6^, ^7^, ^8^ *Oct4, Sox2,* and *Klf4* are considered “pioneer factors” for inducing pluripotency.^8^ Among the pioneer factors, OCT4 protein, encoded by the *Pou5f1* gene, is one of the first to be identified as a master regulator of pluripotency^9^ and is found in both Yamanaka and Thomson cocktails to convert skin cells to iPSCs.^1^^,^^10^ While, recent studies demonstrate that a significant increase in exogenous *Oct4* expression over moderate levels negatively affects reprogramming^11^^,^^12^ and iPSC quality.^13^ Others studies have shown that exogenous *Oct4* expression can be excluded entirely, inducing low efficiency and slower kinetics,^13^ or replaced with other factors in certain conditions.^14^ Altogether, these indicate that the role of exogenous OCT4 in reprogramming remains unclear. To address the question if the initial overexpression of OCT4 or its continuous expression throughout the reprogramming is favorable or detrimental to the process, it is essential to develop a flexible vector system in which *OCT4* expression can be fine-tuned in a timely manner.

MicroRNA (miRNA) are 20–25-nts-long, small, non-coding RNAs that regulate gene expression by binding to a specific “seed sequence” on the target mRNA and either translationally repress or degrade it, controlling several cellular processes.^15^, ^16^, ^17^, ^18^ This regulation system, nicknamed miRNA targeting, is used to restrict (*trans*)gene expression to a particular cell or tissue type by incorporating miRNA target sequence (MTS) in the 3′ untranslated region (UTR) of a specific gene. This process has been exploited in viral vectors to 1) control viral replication or propagation, 2) control virulence of oncolytic virus, 3) eliminate viral transgenes, or 4) to prevent it from regulating its natural targets.^19^, ^20^, ^21^, ^22^, ^23^, ^24^, ^25^, ^26^

MeV is a negative sense, non-segmented, single-stranded RNA virus that belongs to the *Paramyxoviridae* family.^27^ Recombinant MeVs have been used as an oncolytic in clinical settings,^28^ but its application as a viral vector in reprogramming has only started recently.^29^, ^30^, ^31^ MiRNA-controlled oncolytic MeVs have previously been developed to increase tumor specificity and protect the surrounding organs. Specific MTSs were introduced either in the 3′UTR of the F gene to abolish fusion and propagation^32^^,^^33^ or in the 3′UTR of the P, N, or L gene to control replication.^33^^,^^34^ In 2019, our lab developed a single-cycle MeV, by substituting the hemagglutinin gene with GFP or RFs.^29^^,^^30^ The MeV-derived iPSC could re-differentiate into the three germ lineages, and vector-free iPSCs were achieved in 3–5 clonal passages, indicating an excellent future for the single-cycle MeV reprogramming platform.^29^^,^^30^ However, the first generation of MeV reprogrammed with low efficiency and additional effort in vector design was required to bring MeV reprogramming to a competitive level.

Here, we present a more efficient reprogramming MeV vector and a novel way to use miRNA targeting. In this study, we take advantage of the modular gene arrangement in MeV to separate the *OCT4* gene from the *SOX2*-*KLF4* bicistron to increase reprogramming efficiency. We then selected a miRNA, which is specifically upregulated during MET,^35^, ^36^, ^37^ miR-375, and showed that we can use it to control the expression of OCT4 by MET. This direct control of OCT4 increases both the kinetics and efficiency of reprogramming. Through indirect silencing of OCT4, using P gene silencing, we demonstrate that OCT4 is dispensable for the maturation phase of reprogramming, while all three other factors, SOX2, KLF4, and c-MYC, are not. Finally, our study demonstrates a novel application for miRNA targeting in a viral vector in an autoregulatory loop to improve the overall efficiency of the vector-induced process.

## Results

### Production of a MeV vector expressing OCT4 under the control of MTS

**Figure 1:**
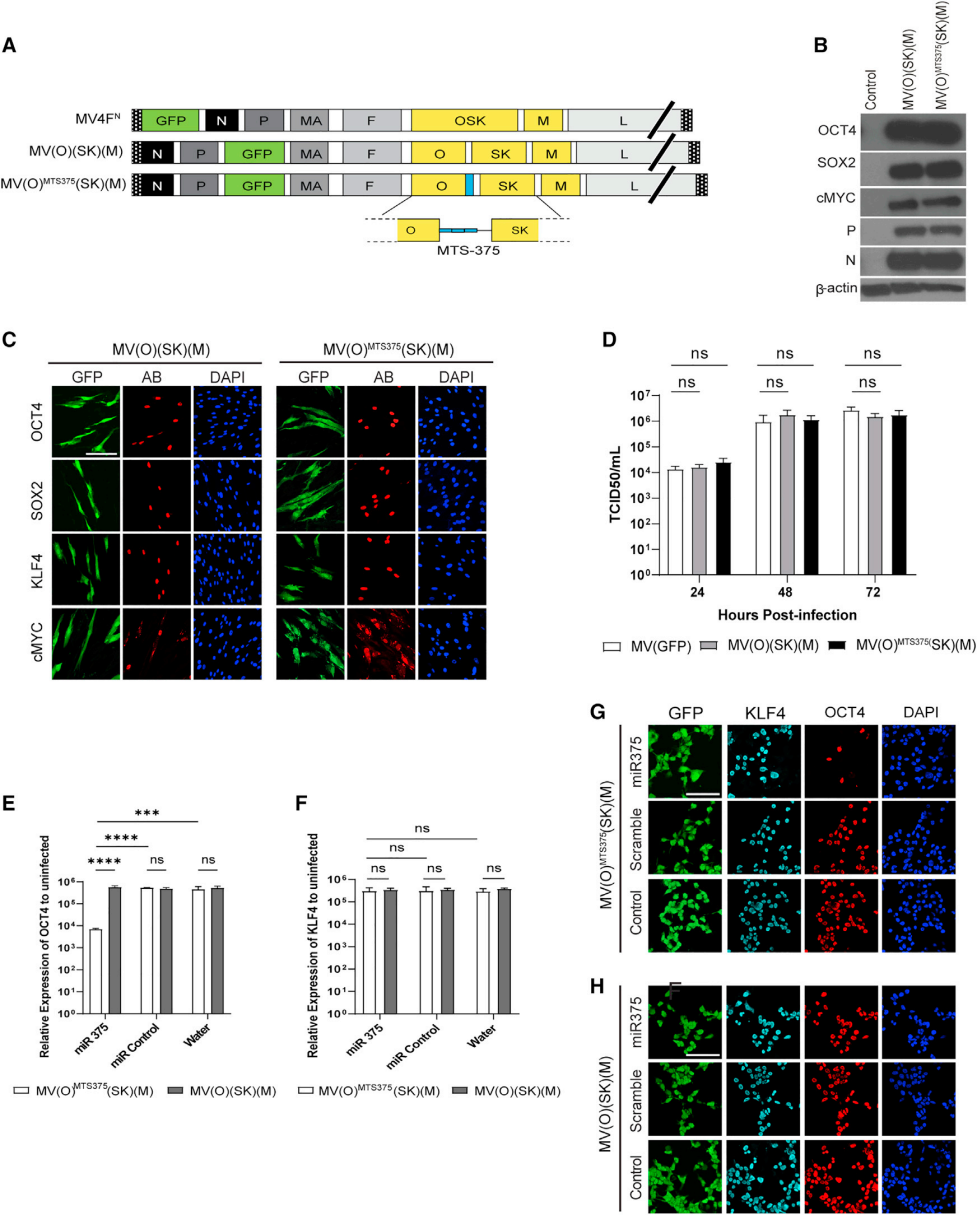
Genome structure and characterization of single-cycle measles vector with MTS in the 3′ UTR of *OCT4* gene (A) Schematic of MeV reprogramming vectors. (B) Western blot analysis of *OCT4*, *SOX2*, and *cMYC* expression in NHFs transduced cells with the indicated vector or control (Uninfected). β-Actin as a loading control and MeV N and P as infection control. (C) Representative confocal images of *OCT4*, *SOX2*, *KLF4*, and *cMYC* nuclear expression in transduced NHF cells with the indicated vector. Scale bars represent 100 μm. (D) One-step growth curves of indicated vectors and control virus on Vero-H2 cells. Data represent values from three independent experiments. Error bars indicate mean ± SD. A two-way ANOVA was used followed by Tukey's multiple comparison test (ns, not significant) (E and F) qPCR analysis of *OCT4* (E) and *KLF4* (F) expression from indicated vectors in cells transfected with miR-375, scrambled miRNA, or water. Data were normalized to *GAPDH* and represent the average ±SD of the mean from three independent replicates. A two-way ANOVA was used followed by Tukey's multiple comparison test (ns, not significant, ∗∗∗p ≤ 0.001 and ∗∗∗∗p ≤ 0.0001). (G and H) Immunofluorescence analysis of *OCT4* (G) and *KLF4* (H) expression from indicated vectors in 293T cells transfected with miR-375, scrambled miRNA, or water. Scale bars represent 100 μm.

We have previously produced a single-cycle MeV expressing the four RFs, MV4F^N^, to produce iPSCs.^29^ MV4F^N^ expresses tricistron *OSK* instead of MeV hemagglutinin (*H*) and *c-MYC* in an additional transcription unit (ATU) after the *H* gene (Figure 1A, top genome). We modified this vector by isolating the *OCT4* from a *SOX2* and *KLF4* bicistron (SK) in an individual ATU to make the MV(O)(SK)(M) vector (Figure 1A, middle genome). In MV(O)^MTS375^(SK) (M), we introduced three copies of an MTS for miR-375 (MTS375) in the 3′ UTR of the *OCT4* gene (Figure 1A, bottom genome). For both, MV(O) (SK)(M) and MV(O)^MTS375^(SK)(M) vectors, GFP was inserted in the ATU after the *P* gene to track the vector. To confirm the expression and correct processing of the RFs from the three individual ATUs, Western blot analysis of neonatal human fibroblasts (NHFs) infected with both vectors was performed (Figure 1B). Expression of OCT4, SOX2, and c-MYC with appropriate molecular weight was observed, indicating that the addition of the MTS375 in the 3′ UTR region of *OCT4* did not affect its expression. Confocal analysis confirmed the nuclear localization of the RFs (Figure 1C). To address the possible effect of an additional ATU on the MeV vector propagation, a one-step growth curve was performed. All vectors showed comparable growth kinetics with the replication-competent MV(GFP) virus and replicated to maximum titers of greater than 10^6^ by 48 h, confirming that vector propagation remains unaltered by the insertion of an additional ATU and the MTS375 in the 3′UTR of *OCT4* (Figure 1D).

Subsequently, we verified that the MTS375 control of *OCT4* by miR-375 was functional. Cells (293T) were initially transfected with either miR-375, scramble miR precursors, or water, followed by transduction with either MV(O)(SK)(M) or MV(O)^MTS375^(SK)(M). Seventy-two hours later, cells were either collected for qPCR analysis (Figures 1E and 1F) or fixed for immunofluorescence and confocal analysis (Figures 1G and 1H). While the relative expression of *OCT4* mRNA from MV(O)(SK)(M)-transduced cells remains unaffected by the presence miR-375 precursors, it significantly decreases in cells transduced with MV(O)^MTS375^(SK)(M) (Figure 1E). Control transfection with scrambled miRNA or water did not affect the level of *OCT4* mRNA in cells transduced either with MV(O)^MTS375^(SK)(M) or MV(O)(SK)(M) (Figure 1E). The level of *KLF4*, expressed from a different ATU, remained unchanged in all conditions, indicating the specificity of the MTS375 silencing of *OCT4* (Figure 1F). Results were validated by confocal analysis (Figures 1G and 1H). Most cells transfected with miR-375 and subsequently transduced with MV(O)^MTS375^(SK)(M) expressed KLF4 but not OCT4 (Figure 1G, top panel). On the other hand, both KLF4 and OCT4 were expressed in all cells transfected with scrambled miRNA or water (Figure 1G, middle and lower panels). Finally, in any conditions, cells transduced with MV(O)(SK)(M) expressed both KLF4 and OCT4 (Figure 1H). Taken together, the results show that the MTS375 control of OCT4 by miR-375 is functional and specific.

### miR-375 is upregulated during the MET phase of human fibroblasts reprogramming

**Figure 2:**
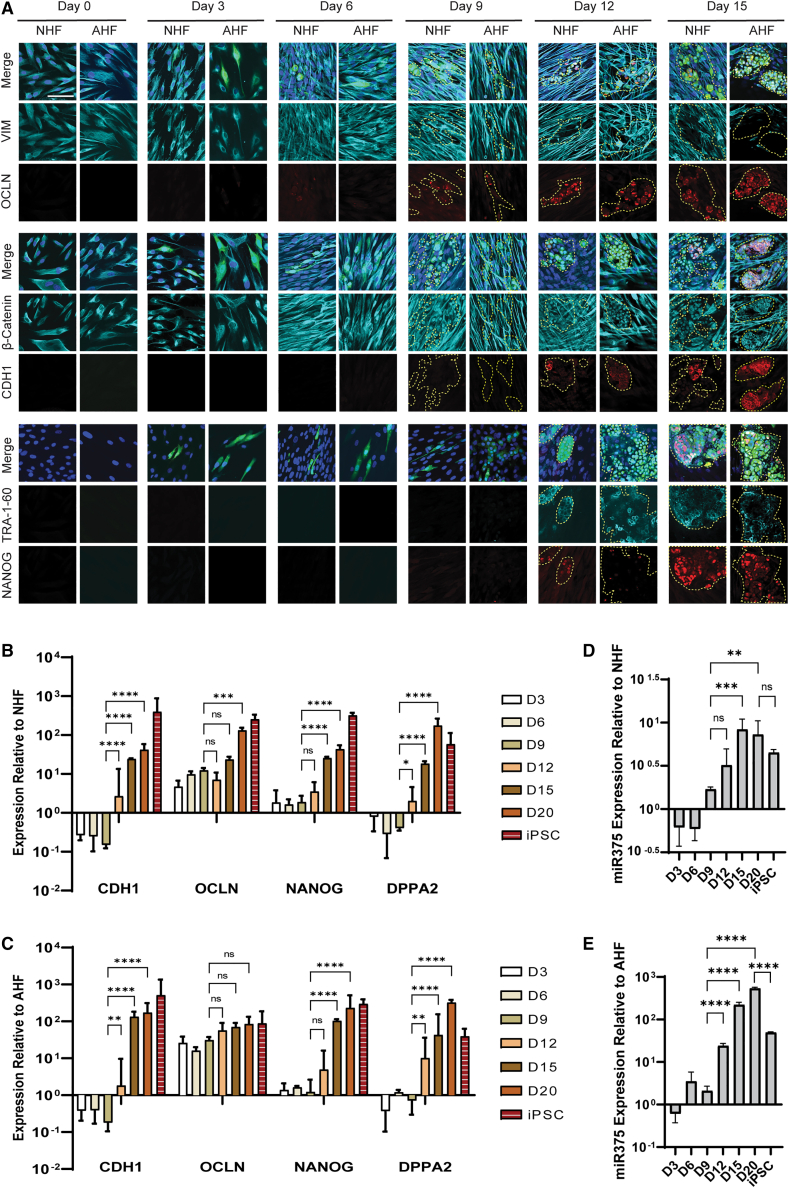
Human fibroblasts complete MET within the first 15 days of MeV reprogramming and miR375 is associated with it (A) Immunofluorescent staining with specified markers at days 0, 3, 6, 9, 12, and 15 of NHF and AHF reprogramming. Merge is presented as overlays of indicated antibody staining, GFP, and Dapi staining. GFP+ cells within areas of interest are highlighted with yellow dashed lines for better visualization. The scale bars represent 100 μm. (B and C) qPCR analysis of specified gene during NHF (B) and AHF (C) reprogramming. All values are relative to day 0 and normalized to *GAPDH*. Error bars represent mean ± SD with n = 3 independent experiments. A two-way ANOVA was used followed by Tukey's multiple comparison test (∗p ≤ 0.05, ∗∗p ≤ 0.01, ∗∗∗p ≤ 0.001, ∗∗∗∗p ≤ 0.0001). (D and E) qPCR analysis of miR-375 during NHF (D) and AHF (E) reprogramming. Error bars represent mean ± SD. Data represent values from three independent experiments. All values are relative to day 0 and normalized to RNU6B. A one-way ANOVA was used followed by Tukey's multiple comparison test (ns, not significant, ∗∗p ≤ 0.01, ∗∗∗p ≤ 0.001, ∗∗∗∗ p ≤ 0.0001).

**Figure 3:**
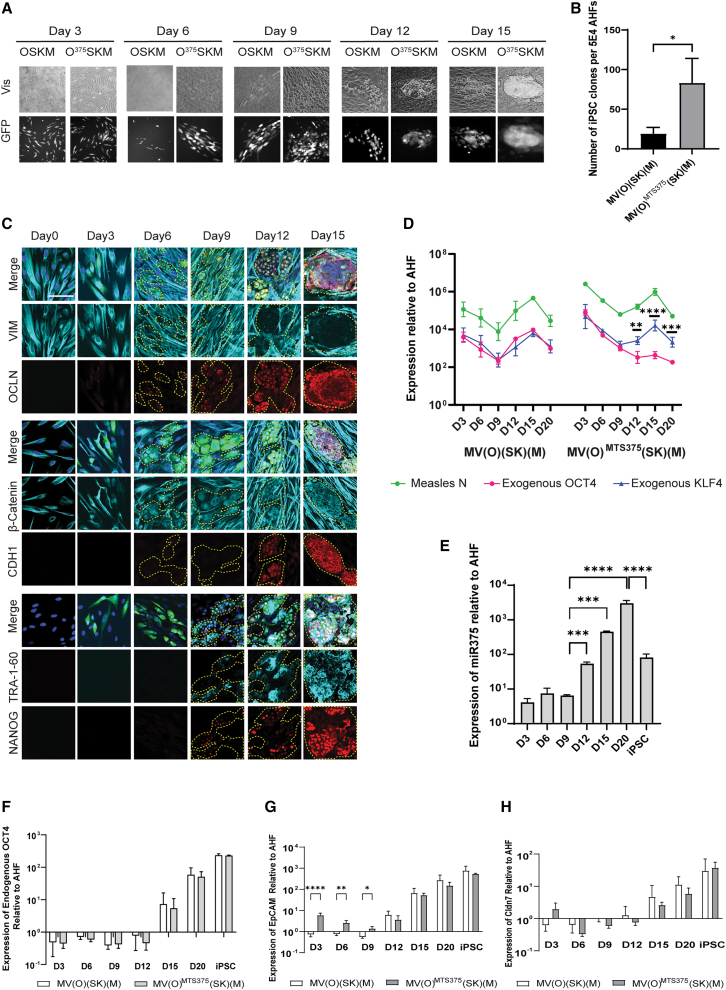
The timely shutdown of exogenous *OCT4* expression by MET improves reprogramming kinetics and efficiency (A) Representative bright-field (top panel) and fluorescent (bottom panel) pictures of AHF reprogramming taken at 3-day intervals at 20× magnifications. (B) Number of TRA-1-60 + iPSC colonies from 5 × 10^4^ AHFs at day 20 of the MV(O) (SK)(M) and MV(O) ^MTS375^(SK)(M) reprogramming. Error bars represent mean ± SD. Data represent values from three independent experiments. Comparison made using unpaired two-tailed T tests (∗p ≤ 0.05). (C) Immunofluorescent labeling of specified markers at days 0, 3, 6, 9, 12, and 15 of AHF reprogramming. Merge is presented as overlays of indicated antibody staining, GFP, and Dapi staining. GFP+ cells within areas of interest are highlighted with yellow dashed lines for better visualization. The scale bars represent 100 μm. (D) qPCR analysis of relative expression of exogenous *OCT4* (pink), *KLF4* (blue), and Measles *N* (green) during AHF reprogramming with indicated vectors. All values are relative to day 0 and normalized to *GAPDH*. Error bars represent mean ± SD with n = 3, independent experiments. A two-way ANOVA was used followed by Sidak post hoc multiple comparisons test to compare the relative expression levels of *OCT4* and *KLF4* (∗∗p ≤ 0.01, ∗∗∗p ≤ 0.001, ∗∗∗∗p ≤ 0.0001). (E) qRT-PCR analysis of endogenous miR-375 expression in MV(O)^MTS375^(SK) (M) reprogramming. All values are relative to day 0 and normalized to RNU6B. Error bars represent mean ± SD. Data represent values from three independent experiments. A one-way ANOVA was used followed by Tukey's multiple comparison test (∗∗∗p ≤ 0.001, ∗∗∗∗p ≤ 0.0001). (F–H) qPCR analysis of indicated endogenous *OCT4* (F), *EPCAM* (G), and *CLDN7* (H) during AHF reprogramming. All values are relative to day 0 and normalized to *GAPDH*. A two-way ANOVA was used followed by Tukey's multiple comparison test. Error bars represent mean ± SD, n = 3, independent experiments (∗p ≤ 0.05, ∗∗p ≤ 0.01, ∗∗∗∗p ≤ 0.0001).

We identify the MET timelines of MeV reprogramming in NHFs and adult human fibroblasts (AHFs) using an MV(O)(SK)(M) vector. Transduced cells were analyzed every 3 days for 15 days by confocal microscopy and until day 20 by qRT-PCR. Mesenchymal (Vimentin), epithelial (Occludin, E-cadherin, β-catenin), and iPSC (NANOG, TRA-1-60, DPPA2) markers were used to determine the different cell states (Figure 2). From day 0 to day 6, Vimentin continued to be assembled into a network of filaments in the cytoplasm in both NHF and AHF (Figure 2A, second row). While MeVs can be detected by day 3 (GFP+ cells), it is only by day 9 that the GFP+ cells gained more epithelial morphology, with downregulation of Vimentin. The loss of Vimentin was synchronized with a weak expression of Occludin, indicating the initial formation of tight junctions. By days 12–15, the loss of Vimentin was evident, and the expression of Occludin stronger, suggesting loss of mesenchymal characteristics and gain of epithelial junctions. Subcellular localization of β-catenin is a marker of MET. By day 9, as GFP+ cells started losing their long protrusions and decreasing in size, a transition of β-catenin from the cytoplasm toward the nucleus was observed (Figure 2A, fourth row). By day 12, some GFP+ cells displayed both nuclear and cellular membrane expression of β-catenin. β-catenin's cortical distribution was reported to help in the formation of adherens junctions in emerging iPSC colonies.^38^ The cortical expression of β-catenin and E-cadherin was observed by day 15 in most of the GFP+ cells, confirming completion of MET. Finally, expression of pluripotency markers NANOG and TRA-1-60 were observed starting at day 12 and onwards for both NHFs and AHFs (Figure 2A, eighth and nine rows). Confocal approach was validated by showing establishment of MET between days 6 and 9 in NHF lentivirus (LV)-mediated reprogramming (Figure S1), as previous reported.^39^, ^40^, ^41^ The establishment of MET in MeV-mediated reprogramming was also verified using RT-qPCR in NHFs and AHFs (Figures 2B and 2C). E-cadherin, Occludin, and EPCAM's relative expression increased between days 9 and 12 and onwards, supporting that MET was initiated by day 9 (Figures 2B, 2C, and 3G). Expression of pluripotency markers, NANOG or DPPA2, started by days 12–15 (Figures 2B and 2C). On the other hand, there was no significant decrease in the expression of Vimentin (Figure S2), and this is mostly due to the presence of a high number of untransduced fibroblasts.^6^ Taken together, these results indicate that MET occurs between days 9 and 15 and is also cell maturity independent, as it occurs at a similar time frame in both NHFs and AHFs.

Previous reports demonstrated that miR-375 enhances MET in various cancer models and β cell-derived cells.^35^, ^36^, ^37^ However, there is no existing report on the expression or involvement of miR-375 during reprogramming, making it an ideal candidate to regulate exogenous *OCT4* without affecting reprogramming process. We determined the kinetics of miR-375 expression during MeV reprogramming of NHFs and AHFs using RT-qPCR analysis. While more modest in NHFs, a significant upregulation of miR-375 was observed in both NHFs and AHFs starting between days 9 and 12 and onwards. In both cells, the upregulation was directly correlated with the timing of the MET.

### The timely shutdown of exogenous *OCT4* by MET improves reprogramming kinetic and efficiency

To evaluate the kinetics and efficiency of the MV(O)^MTS375^(SK)(M) vector over the established MV(O) (SK)(M), reprogramming with both vectors was conducted in parallel. Reprogramming kinetics were visually assessed by tracking GFP+ cells in 3-day intervals, and cells were stained for TRA-1-60 on day 20 (Figures 3A and 3B). On day 3, the transduced MV(O)^MTS375^(SK)(M) fibroblasts retained their mesenchymal structure with long protrusions, but by day 6, clusters of GFP+ cells started showing circular morphology. By day 9, GFP+ cells from MV(O)^MTS375^(SK)(M) reprogramming lost their fibroblast protrusions and gained epithelial morphology by day 12, then progressed to form iPSC-like clones, which matured by day 15. In contrast, in MV(O)(SK)(M) reprogramming, the GFP+ cells gained epithelial morphology only around day 12 before proceeding to form small iPSC-like clusters around day 15, indicating that the MV(O)^MTS375^(SK)(M) reprogramming kinetics are about 3 days faster to MV(O)(SK)(M), in either AHFs or NHFs (Figure 3A and not shown). Reprogramming efficiencies with MV(O)^MTS375^(SK)(M) showed a significant 3- to 5-fold increase compared with MV(O)(SK)(M) (Figure 3B and not shown), indicating reprogramming was also increased by the control of *OCT4* by miR-375.

We next evaluated the influence of the presence of the MTS375 in the 3′ UTR of *OCT4* on the MET timelines (Figures 3C and S3A). Starting at day 6, a weak signal for Occludin and a loss of Vimentin were observed, and this became more significant as time progresses by days 9 and 12 for AHF reprogramming (Figure 3C, second and third rows, and Figure S3D). Similar results were observed on NHFs (Figures S3A and S3C). While the subcellular localization of β-catenin followed the same pattern as previously described for MV(O)(SK)(M) reprogramming; once again, these processes started at day 6 instead of day 9 (Figures 3C and S3A, fifth row). However, there was no change in the timing of the expression of E-cadherin between both vectors at day 12, and the subsequent increase in its expression is comparable (Figure 3C, sixth row, compare with Figures 2A and S3D). Similar results were observed in NHFs (Figures S3A and S3C). These results indicated an early beginning to the formation of the tight junctions and confirmed that MET occurred 3 days faster in MV(O)^MTS375^(SK)(M) reprogramming. Finally, the expression of pluripotency markers NANOG or TRA-1-60 was not uniform in all clones. Unlike MV(O)(SK)(M) reprogramming, where no clones exhibited their expression at day 9, around 20% of the clones in MV(O)^MTS375^(SK)(M) showed early expression of TRA-1-60 and NANOG (Figure 3C, eight and nine rows, compare with Figure 2A). However, at the transcript level, there was no significant difference in NANOG expression between MV(O)(SK)(M) and MV(O)^MTS375^(SK)(M) (Figure S3D, compare with Figure 2C). Similar results were observed on NHF (Figures S3A and S3C).

We next determined the status of the exogenous and endogenous *OCT4* during reprogramming. Specific primers for either the codon-optimized exogenous or endogenous *OCT4* were used to differentiate between both transcript populations. While the expression kinetics pattern for exogenous *KLF4* mRNA followed the MeV nucleoprotein (*N*) mRNA, there was a gradual loss in exogenous *OCT4* expression during MV(O)^MTS375^(SK)(M) reprogramming (Figure 3D, right half). This gradual loss was directly correlated with the increase in miR-375 (Figure 3E). In contrast, during MV(O)(SK)(M) reprogramming, all three mRNA levels, for exogenous *OCT4*, *KLF4*, and *N,* followed a similar expression kinetics (Figure 3D, left half). Expression of *N, OCT4*, or *KLF4* was undetectable in both iPSCs (data not shown). This is attributed to both the addition of the antiviral at D20 and to the known elimination of our single cycle measles vector from iPSC reported in Wang et al.^29^ Additionally, these vectors do not produce permanently infected iPSC, in contrast to the infectious measles virus that can lead to persistent infection.^42^ Interestingly, the overall kinetics of the activation of endogenous *OCT4* remained unaffected by the early elimination of exogenous *OCT4* throughout reprogramming (Figure 3F).

Recent studies showed that Claudin 7 (CLDN7)-mediated modulation of EPCAM, a transmembrane glycoprotein, results in the nuclear translocation of its intracellular domains (EpICD) by forming a complex with β-catenin and FHL2 to regulate gene expression of endogenous *OCT4* and *c-MYC*.^43^, ^44^, ^45^, ^46^, ^47^ Keeping this in mind, we next investigated the expression patterns of EPCAM and CLDN7 using qPCR. While EPCAM was significantly upregulated during early stages in MV(O)^MTS375^(SK)(M) reprogramming compared with MV(O)(SK)(M) (Figure 3G, days 3 and 6), there was no increase in CLDN7 (Figure 3H).

The capability of iPSC-like clones to self-renew, proliferate, and differentiate was tested using confocal microscopy and RT-PCR analyses on isolated individual clones. All pluripotency markers were expressed in all clones tested, and there was no difference in the expression between the clones issued from both vectors (Figures S4A and S4B). The multi-lineages propensity of all clones was confirmed by the formation of the embryoid body and spontaneous differentiation into mesoderm (CD31) endoderm (FOXA2) or ectoderm (β-III tubulin) (Figure S4C, left, middle, and right panel, respectively).

### Embryonic stem-cell-specific miRNA are key players in reprogramming efficiency and kinetics of MV(O)^MTS375^(SK) (M)

**Figure 4:**
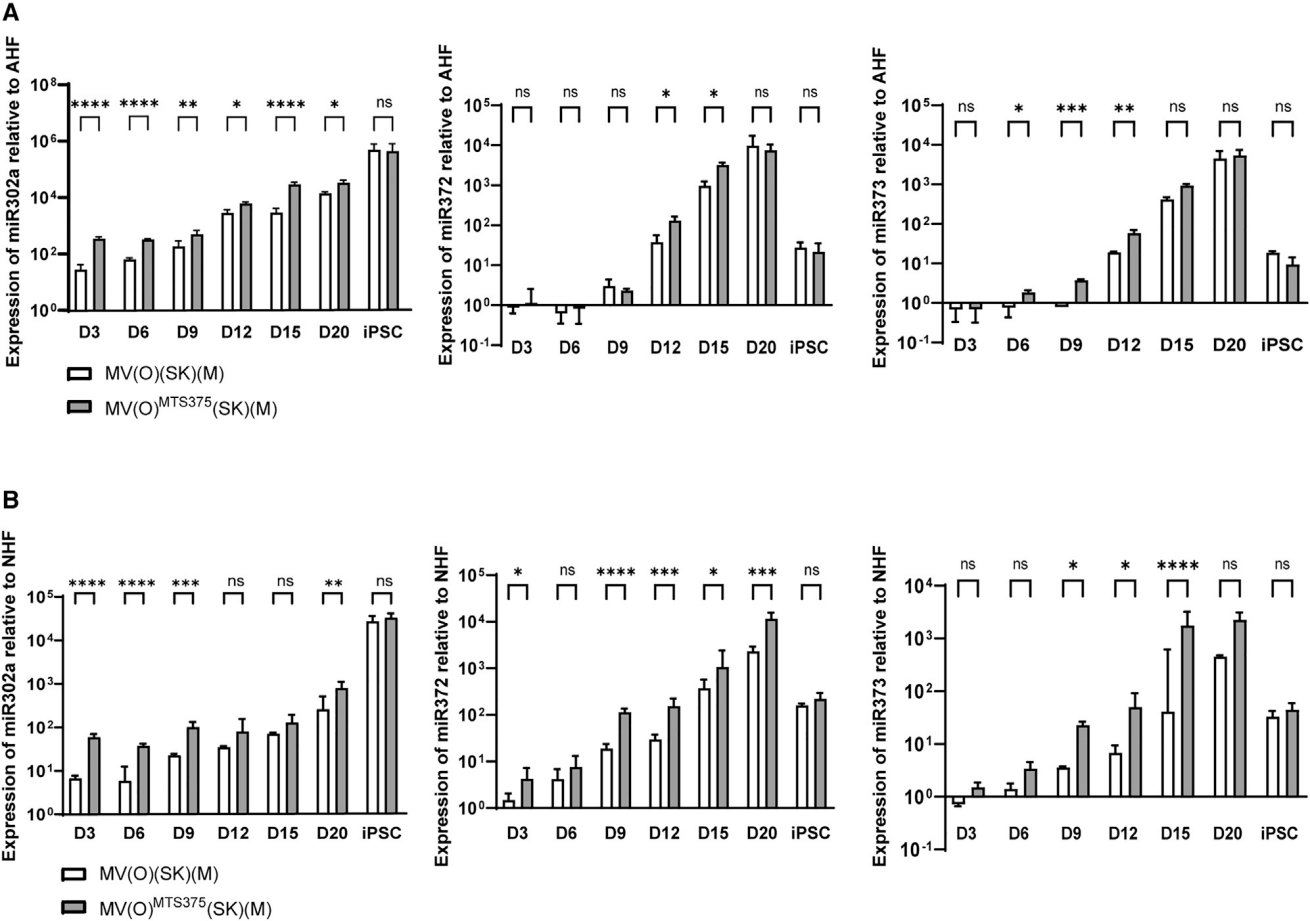
ESCC-specific miRNA are upregulated during MV(O)^MTS37^^5^(SK)(M) reprogramming Fold increase in the endogenous miR-302a left, miR-372 middle, and miR-373 populations during reprogramming of NHFs (A) and AHFs (B) determined by qRT-PCR. Values normalized to RNU6B followed by normalization to day 0. Error bars represent mean ± SD, n = 3, independent experiments. A two-way ANOVA was used followed by Sidak post hoc multiple comparisons test (∗p ≤ 0.05, ∗∗p ≤ 0.01, ∗∗∗p ≤ 0.001, ∗∗∗∗p ≤ 0.0001).

The embryonic stem-cell-specific (ESCC) miRNAs are a group of miRNAs highly expressed in embryonic stem cells and promote cell cycle progression.^48^ We analyzed the expression profiles of miRNAs from the miR-302-367 and miR-371-373 clusters during MV(O)(SK)(M) and MV(O)^MTS375^(SK)(M) reprogramming in both AHF and NHF (Figures 4A and 4B) using RT-qPCR. While an increase in miR-302a expression was observed, starting from the initiation stage (day 3) and onward with MV(O)(SK)(M) in both AHFs and NHFs (Figures 4A and 4B, left panels), the upregulation of miR-372 and miR-373 was synchronized with MET (between days 9 and 15, Figures 4A and 4B, middle and right panels). When cells were transduced with MV(O)^MTS375^(SK)(M), a significant and robust increase in expression of all miRNA was observed at all stages (Figure 4, all panels, top and bottom), indicating a significant upregulation of the ESCC miRNAs that are known to play an essential role in the reprogramming process.

### Shutting down *SOX2*, *KLF4*, and *c-MYC* by MET is detrimental to MeV reprogramming

**Figure 5:**
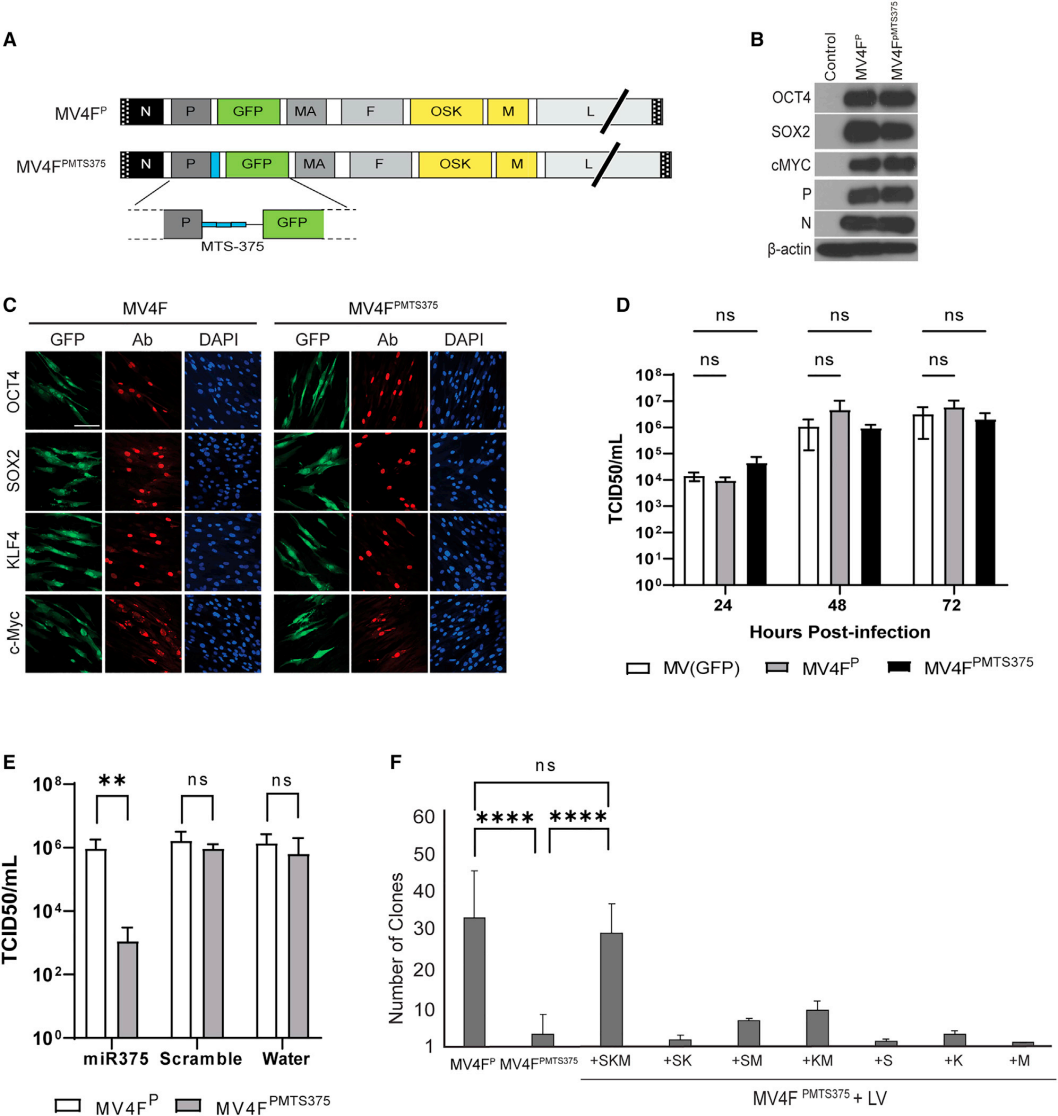
Shutting down *SOX2*, *KLF4*, and *c-MYC* by MET is detrimental to MeV reprogramming (A) Schematic of MeV reprogramming vectors. (B) Immunoblot analysis of *OCT4*, *SOX2*, and *cMYC* expression on BJ cells. Uninfected BJ (control), β-Actin (loading control), and MeV N and P (infection control). (C) Representative confocal images of *OCT4*, *SOX2*, *KLF4*, and cMYC nuclear expression in transduced NHF cells with the indicated vector. Scale bars: 100 μm. (D) Titers of cell-associated and released virus produced upon infection of Vero-H2 cells with MV4F^P^ and MV4F^P^^MTS^^375^ compared with MV(GFP). Error bars represent SD. Data from three individual experiments. A two-way ANOVA was used followed by Tukey's multiple comparison test (ns, not significant). (E) Virus growth kinetics of indicated vector in cells transfected with miR-375, scrambled miRNA, or water. Thirty-eight hours post-infection, cells were scraped in their medium. Virus progeny was tittered on Vero-H2 cells. Error bars represent SD of three technical replicates (n = 3). (F) Number of iPSC colonies from 7 × 10^4^ NHFs at day 20 of the specified MeV or MeV + LV reprogramming. NHFs were either transduced with MV4F^P^/MV4F^P^^MTS^^375^ vector or co-transduced with MV4F^P^^MTS^^375^ vector and individual LV vectors carrying *SOX2* (S), *KLF4* (K), or *c-MYC* (M). Error bars represent mean ± SD. Data represent values from three independent experiments. Comparison made using unpaired two-tailed T tests (∗∗∗∗p ≤ 0.0001).

We next explored the effect of eliminating *SOX2*, *KLF4*, and *c-MYC* by MET during MeV reprogramming. Two additional vectors MV4F^P^ and MV4F^PMTS375^ were produced. MV4F^P^ expresses triciston *OSK* instead of *H* and *c-MYC* in the ATU after the tricistron (Figure 5A, top genome). MV4F^PMTS375^ was modified by inserting three repeats of the MTS375 in the 3′ UTR of *P* gene (Figure 5A, bottom genome). P protein of MeV serves as a cofactor to the virus polymerase L and plays a major role in replicating and transcribing the vector.^34^^,^^49^, ^50^, ^51^ Hence, targeting the MeV P using miRNA will control viral replication and transcription and, with it, the expression of all four RFs.

The expression of the RFs, OCT4, SOX2, and c-MYC, as well as the expression of the viral proteins, were not affected by the presence of the MTS375 in the 3′UTR of the *P* gene (Figure 5B), indicating that the presence of the MTS375 did not affect the cofactor function of the P. Confocal microscopy confirmed the nuclear localization of the RFs in human fibroblasts transduced with both vectors (Figure 5C). To determine the effect of MTS375 insertion on vector propagation, we performed a one-step growth curve analysis on the MV4F^P^ and MV4F^PMTS375^ and compared it with a replication competent MV(GFP) virus. All vectors replicated to comparable titers, indicating that the insertion of the MTS375 in the 3′UTR of the *P* gene does not affect vector replication and propagation (Figure 5D). We next determined the functionality of the MTS375 insertion on the 3′UTR of *P* gene (Figure 5E). Cells (293-H) expressing the MeV *H* gene were transfected with miR-375 or scrambled miRNA precursors or water and later transduced with either MV4F^P^ or MV4F^PMTS375^ vectors. Propagation of MV4F^PMTS375^ was significantly decreased in the presence of miR-375, as shown by the reduction in titers and syncytia formation, compared with cells treated with control miRNA or water (Figures 5E and S5). Unlike MV4F^PMTS375^, MV4F^P^ replicated to high titers in all conditions (Figures 5E and S5), showing that the targeting of P by miR-375 is functional.

We next analyzed the ability of MV4F^P^ and MV4F^PMTS375^ vectors to reprogram NHFs. While the MV4F^P^ vector reprogrammed NHFs, the efficiency of MV4F^PMTS375^ was drastically decreased (Figure 5F). To determine if this effect was due to the early elimination of all exogenous RFs by MET, reprogramming of MV4F^PMTS375^ was performed in the presence of individual LV-expressing *SOX2*, *KLF4*, and *c-MYC*. This LV co-transduction ensured the expression of SKM after MET, while OCT4 expression was suppressed through MTS375 targeting. Reprogramming with MV4F^PMTS375^ was rescued by adding the three LVs and reached similar efficiencies than MV4F^P^. However, transduction with a combination of one or two LVs could not rescue MV4F^PMTS375^ reprogramming (Figure 5F), indicating that exogenous *OCT4* can be shut down by MET but not *SOX2*, *KLF4*, and *c-MYC* (Figure 5F).

The self-renewal, proliferation, and differentiation ability of the iPSC-like clones derived from MV4F^P^ and MV4F^PMTS375^ + LV were performed using immunofluorescence or RT-PCR on isolated individual clones. All clones tested expressed the pluripotency markers, and there was no difference in the expression between the clones issued from both vectors (Figures S6A and S6B). Multi-lineage propensity was established by the formation of embryoid body and spontaneously differentiating them into mesoderm, endoderm, or ectoderm (Figure S6C), indicating that all clones produce are iPSCs.

## Discussion

This study demonstrates an autoregulatory system that amplifies the efficiency of the process initiated by the vector. We validate a new application for miRNA targeting in a viral vector that activates a vector-induced miRNA, which in turn controls the vector itself or one of the transgenes in an autoregulatory loop, resulting in increased efficiency of the process started initially by the vector. In this case, the MeV vector expressing the four RFs starts the reprogramming process and induces miR-375. Mir-375 can then bind to the MTS375 in the 3′UTR of the *OCT4* gene expressed from the vector, causing its elimination during the MET phase, increasing reprogramming efficiency. In this work, we further demonstrate that only *OCT4*, and not SKM, is dispensable for the maturation stage of reprogramming.

Previous studies using partially and intermediately reprogrammed iPSC have facilitated context-dependent studies of reprogramming.^52^, ^53^, ^54^ However, their production is sporadic and unpredictable, since reprogramming is transient and asynchronous.^55^^,^^56^ Unlike the previous methods, the miRNA controllable MeV vector system allows for context-dependent expression of RFs at a single cell level, catering to the asynchronous nature of reprogramming. Additionally, as MeV vector replicates in the cytoplasm, it is free of positional effects, unlike lentivectors.^57^ Another hurdle in cell reprogramming is the low efficiency and slow kinetics of iPSC production. Many modulators (miRNA mimics and small molecules) have been identified to enhance iPSC generation.^58^ In comparison with other strategies to enhance efficiency, the main advantage of this technology is simplicity. We showed that the expression of OCT4 can be fine-tuned in MeV by exploiting endogenous miRNA to ensure a precise and optimized level of transgene to improve efficiency. Hence, in this study, the new and improved MeV vector can reprogram AHFs with an average efficiency of 0.16% compared with 0.046%. Further, our novel approach could be incorporated into existing reprogramming systems.

During the initiation phase, the RFs, *OCT4*, *SOX2*, and *KLF4*, act as “pioneer factors” and bind to the inaccessible chromatin regions, leading to subsequent activation or repression of genes.^8^ However, their role during the maturation phase is not well understood. We demonstrate that with the exception of OCT4, the other RFs (SOX2, KLF4, and c-MYC) are important for iPSC production, even in the maturation phase. This is in accordance with the previous reports that show the dependency of reprogrammed cells on transgene expression during early maturation for survival and growth.^59^ However, silencing of the RFs during later stages of reprogramming is crucial to establish pluripotency.^59^^,^^60^ In MeV reprogramming systems, the RF expression remains high, even during the maturation phase. It is most likely that this high expression of the RFs, especially *OCT4*, is detrimental for the transduced cell to complete reprogramming. By silencing the exogenous OCT4 promptly, by MET, we have allowed more cells to complete the process and adopt iPSC phenotype successfully.

We attribute the increase in reprogramming efficiency and the kinetics of MV(O)^MTS375^(SK)(M) to an early expression of EPCAM and ESCC miRNA-miR-302a, miR-372, and miR-373. Indeed, an increase or decrease in EPCAM and ESCC miRNA expression has been shown to improve or decrease the reprogramming efficiency individually.^43^^,^^45^^,^^48^^,^^61^^,^^62^ We believe that early EPCAM expression could be contributing to the faster kinetic of reprogramming by recruiting CLDN7 to the cell-cell junctions.^63^ Further, CLDN7-mediated modulation of EpCAM could result in EpICD (intracellular domain) release, which complexes in the nucleus with proteins of the Wnt signal pathway (β-catenin, Lef-1), initiating gene transcription, leading to an early MET. Additionally, ESCC miRNA, by either targeting nuclear receptor subfamily 2 through indirect positive regulation of *OCT4* or inhibiting the TGF-β signaling pathway, synergistically enhance MET and reprogramming of MV(O)^MTS375^(SK) (M).^61^

Our work demonstrates a novel application of miRNA targeting and that shutting down the expression of exogenous *OCT4* by MET increases the reprogramming efficiency and kinetics. Studying the effects of the temporal expression of the other exogenous RFs on the reprogramming process using the MeV vector systems and miRNAs upregulated during reprogramming could yield insights that can further enhance the efficiency and/or quality of reprogramming.

## Materials and methods

De-identified human cells in this study were either obtained from the American Type Culture Collection (ATCC) or approved through Mayo Institutional Review Board. The viral vectors, viruses, and experiments associated with them were permitted by the Mayo Clinic Institutional Biosafety Committee.

### Cell culture

De-identified human cells AHFs and NHFs were obtained from healthy donors and ATCC (#CRL 2522), respectively. Fibroblasts were maintained in DMEM with 10% ES-FCS (Life Technologies, Carlsbad, CA, USA), 0.1mM non-essential amino acids (Corning Mediatech, Manassas, VA, USA), and 1% Penicillin and Streptomicin (P/S, Corning Mediatech, Manassas, VA, USA) (media 1). During reprogramming, iPSCs were cultured in 80% Nutristem hPSC XF medium (Biological-Industries, Kibbutz Beit-Haemek, Israel) with 20 ng/mL of human recombinant bFGF (STEMCELL Technologies, Vancouver, Canada), 20% mTeSR1 (STEMCELL Technologies, Vancouver, Canada), and 1% P/S (media 2). Mature iPSC are maintained in mTeSR1 media (STEMCELL Technologies, Vancouver, Canada). HEK293T, Vero, Vero-H2, helper 293-3-46-H2 and 293LVH cells 29, 30 were cultured in DMEM- with 10% FCS and 1% P/S (DMEM-10). 1.2 mg/ml of G418 was added to the media for the culture of the Rescue-H2 cells (Cardinal Healthcare, Dublin, OH, USA). All cell lines were maintained at 37°C with 5% CO_2_.

### Full-length measles virus cDNA plasmid production

Full-length cDNA vector p(+) MVvac2 ΔH(O)(SK)(cM)H was produced by splitting the one ATU containing the *OSK* polycistron in two ATUs containing the (O) and (SK) bicistron. Addition of the new ATU was performed using PCR and addition of the unique restriction site between the two ATUs to facilitate the cloning. All cloning steps were performed in accord with the “rule of six,” using the intermediate vector pCG containing a PacI-SpeI fragment from full-length p(+) MVvac2ΔH(OSK) (cM)H30 (MV, Figure 1A). Then, the PacI-SpeI fragment was cloned back into the MV full-length genome containing a GFP in an ATU between the P and M gene. The resulting full-length vector was called p(+) MVvac2(GFP)PNΔH(O)(SK)(M)H [MV(O)(SK)(M)] (Figure 1A). Full-length cDNA vector p(+) MVvac2ΔH(O)^MTS375^(SK)(M)H was produced by addition of three target sequences of the miR-375 in the 3′UTR region of the *OCT4* gene using the two following pairs of primers 5′-gcaacgtgctggttattgtgc-3′ and 5′-tcggctcgcgtgaCCATGGtttgttcgttcggctcgcgtgaatttaaaTTAGTTGCTGTGCATTG-3′ and 5′-gaacgaacaaaCCATGGtcacgcgagccgaacgaacaaaCAGTCGtcacgcgagccgaacgaacaaagCTAGcTACAACCTAAATCCA-3′ and 5′-ggagggtaggctagtGGGTATGCC-3′ and amplification of two fragments encoding the *OCT4* and *SOX2-KLF4* fragments and a three-way ligation using the PacI, SalI, and HindIII restriction site in the intermediate pCG-(O)(SK)(M) vector. The SalI restriction site being introduced between the first and second repeat of the miR-375 target sequence. All intermediate and final full-length vectors were fully sequenced to verify the integrity of the vectors. Full-length cDNA vector p(+) MVvac2(GFP)PΔH(OSK)(cM)H was produced by transferring a SfiI-NarI containing the N to M fragment of the p(+)MVvac2(GFP)P vector. To obtain the full-length cDNA vector p(+) MVvac2(GFP)P^MTS375^ΔH(OSK)(cM)H, we first produced a p(+)MVvac2(GFP)P^MTS375^ΔH(GFP)H vector by adding three target sequences of the miR-375 in the 3′UTR region of the P gene using the two following pairs of primers 5′-ctcagcaattggatcaac-3′ and 5′-cgcgtgacCATGGtttgttcgttcggctcgcgtgaGGTTGGCAGGTAAGTTG-3′ and 5′-gaacaaaCCATGgtcacgcgagccgaacgaacaaaCAGTCGtcacgcgagccgaacgaacaaaACCCAactagcctaccc-3′ and 5′-agcctgccatcactgta-3′ and amplification of two fragments encoding the *P* and *GFP* fragments and a three-way ligation using the SacII, NcoI, and BssHII restriction sites in the p(+)MVvac2(GFP)PΔH(GFP)H vector. Then, a SpeI-PacI fragment containing the ΔH(OSK)(M)H fragment was cloned instead of the ΔH(GFP)H, producing the final vector p(+) MVvac2(GFP)P^MTS375^ΔH(OSK)(cM)H. All intermediate and final full-length vectors were fully sequenced to verify the integrity of the vectors.

### Viral vector production

Rescue of recombinant MeV vectors from transfected plasmids was carried out, as previously described, using Vero-H2 and helper 293-3-46-H2 cells.^30^ In brief, the MV genome and MV polymerase were co-transfected into the helper 293-3-46 H2 cells and were transferred onto Vero-H2 cells 3 days later. The rescue was monitored for appearance and spread of GFP, and the virus was further expanded on Vero-H2 cells. All vectors were propagated on Vero-H2 cells, and stocks from the second or third passage were used for all experiments. Virus titers were determined by titration on Vero-H2 cells, by 50% endpoint dilution (TCID50) with individual infection events counted by GFP fluorescence at 72 h post infection using the Spearman-Kärber method.^64^

### Reprogramming of human fibroblasts cells

NHF (7 × 10^4^) and AHF cells (5 × 10^4^) were seeded on Matrigel (Corning, Corning, NY, USA)-coated 12-well plates. Cells were either transduced with MeV vectors alone (MOI of 0.5) or in combination with LV vectors (at best optimal volume).^30^ The virus and cells were spinoculated at 1,100 rpm for one hour at 25°C, after which the inoculum was left O/N at 37°C. The next day, infected cells were washed, and media 1 was added. Subsequently, the media were changed every other day until day 8, after which it was switched to media 2, with daily replacement until iPSC-like clones appear around days 20–25. For AHF, the cells were split onto two 6-well Matrigel-coated plates in media 1. On day 7, and for the next 7 days, cells were switched to media 2 containing small molecules (sm); SB431542 (5μM), PD0325901 (0.2μM), and Thiazovivin (0.5μM) (All Stemgent, Cambridge, MA, USA). After day 14, daily media change was performed with media 2 without sm, until iPSC-like clones were ready to be picked or fixed. At day 20, anti-MeV inhibitor, AS-136A (Sigma Aldrich, St. Louis, MO, USA), was added to eliminate the viral vector. Reprogramming efficiency was calculated as the percent of TRA-1-60-positive iPSC colonies generated divided by the number of input cells. TRA-1-60-positive colonies were visualized with NovaRED HRP substrate (Vector Laboratories, Burlingame, California, USA), according to the manufacture.

### One-step growth curves

Vero-H2 cells (4 × 10^5^) were infected with MeV vectors or MeV control virus with an MOI of 0.05 in OptiMEM for two hours at 37°C. Following viral adsorption, the inoculum was aspirated, cells were washed, and DMEM-10 was added. Both cells and supernatant were collected (24, 48, and 72 h post infection), and viral progeny was tittered, as described above.

### Immunostaining and confocal microscopy

For immunostaining, cells on Thermo Fisher Scientific Lab-Tek chamber slides (Sigma-Aldrich, St. Louis, MO, USA) or a 12-well tissue culture plate (MatTek corporation, Ashland, MA, USA) were fixed with 4% PFA, permeabilized, and stained for appropriate primary antibodies in 5% FBS/PBS O/N at 4°C and corresponding secondary antibodies for 1 h at room temperature (RT). Once stained, they were mounted with Prolong Gold Antifade (Life Technologies, Carlsbad, CA, USA). Further analyses were performed using a Zeiss LSM 780 confocal microscope followed by image processing with Zen black software (Zeiss). Primary and secondary antibodies are listed in Table S1.

### Western blot

BJs (2.1 × 10^5^) were transduced with MeV at MOI 0.5. Cells were processed according to previously described procedures, following 36 h.^30^ Separation of protein samples was performed on SDS page gels (Bio-Rad Laboratories, Hercules, CA, USA), followed by a transfer to polyvinylidene difluoride membranes (Immobilon-P, Bio-Rad Laboratories, Hercules, CA, USA). After blocking the membranes, they were incubated with primary antibodies. Following washes, membranes were subjected to peroxidase-conjugated secondary antibodies for 2 h at room temperature. Three washes later with TBS-Tween 0.1%, membrane was subjected to ECL2 substrate (Thermo Pierce, Waltham, MA, USA). Table S2 lists the primary and secondary antibodies.

### Cellular and viral gene transcription by qRT-PCR and RT-PCR

Cells were subjected to Trizol reagent (Life Technologies, Carlsbad, CA, USA) to extract total RNA. EcoDry TM Premix Oligo dT kits (Takara Bio, Shiga, Japan) were used for cDNA synthesis. The transcript level of the target gene was determined using TaqMan PreAmp Master Mix (Thermo Fisher Scientific, Waltham, MA, USA). The gene expression was normalized to *GAPDH* and was calculated using the delta-delta-Ct algorithm. Platinum Taq DNA polymerase (Life Technologies, Carlsbad, CA, USA) was used to amplify the cDNA, subsequently used for PCR. Primers and probes are listed in Table S3. RT-PCR to verify pluripotency markers in iPSC clones was carried out using Platinum Taq DNA polymerase (Life Technologies, Carlsbad, CA, USA). Primers for pluripotency markers were described in previous literature.^30^

### miRNA targeting assay

HEK293LV-H cells or HEK293T cells (2 × 10^5^) were seeded in 12-well Matrigel plates. The next day, we transfected miRNA mimics (*mir*Vana miRNA Mimics, Life Technologies, Carlsbad, CA, USA) at a final concentration of 40 nM using Lipofectamine 2000 transfection reagent (Life Technologies, Carlsbad, CA, USA). Cells were transduced with MeV vectors at an MOI of 0.05, 4 h post-transfection. Viral vectors inoculum was incubated with the cells for 2 h at 37C, later removed, followed by a wash, and media addition. To determine production of virus, transduced cells were scraped, and samples were tittered or qPCR analyzed, as described above. For confocal analysis, HEK293T cells were fixed and immunostained, as described above.

### Spontaneous differentiation assay

Briefly, the iPSCs were detached using EZ-LiFT Stem Cell Passaging Reagent (Sigma-Aldrich, St. Louis, MO, USA). After which, the cells were cultured to form EBs in a non-adherent 6-well plate and subsequently differentiated on Matrigel-coated chamber slides (LABTECKR -II, Thermo Fisher Scientific, Waltham, MA, USA). Subsequently, the cells were subjected to immunostaining and confocal microscopy (as described above) to verify that the iPSCs can differentiate in endoderm (FOXA2), ectoderm (β-III tubulin), and mesoderm (CD-31) lineages. Primary and secondary antibodies are listed in Table S1.

### Statistics

Data were processed in Microsoft Excel. GraphPad Prism 9 was used to graph as well as perform statistical analysis. Single comparisons were made using unpaired two-tailed T tests. Several comparisons were analyzed by one-way or two-way ANOVA, following which Sidak or Tukey's multiple comparison test was used. For reprogramming, growth curve, and transfection/transduction, all experiments are presented as the average of three independent experiments. For qRT-PCR, results are presented as technical triplicate of three independent experiments. Data are graphed as group mean ± SD. Statistical significance cut off at p ≤ 0.05 and ns > 0.05 for all experiments.

## Supplemental information

Document S1. Figures S1–S6 and Tables S1–S3

Document S2. Article plus supplemental information

## References

[1] Takahashi K., Yamanaka S. (2006). Induction of pluripotent stem cells from mouse embryonic and adult fibroblast cultures by defined factors. Cell.

[2] Haridhasapavalan K.K., Borgohain M.P., Dey C., Saha B., Narayan G., Kumar S., Thummer R.P. (2019). An insight into non-integrative gene delivery approaches to generate transgene-free induced pluripotent stem cells. Gene.

[3] Hu K. (2014). All roads lead to induced pluripotent stem cells: the technologies of iPSC generation. Stem Cells Dev..

[4] Li R., Liang J., Ni S., Zhou T., Qing X., Li H., He W., Chen J., Li F., Zhuang Q. (2010). A mesenchymal-to-epithelial transition initiates and is required for the nuclear reprogramming of mouse fibroblasts. Cell Stem Cell.

[5] Samavarchi-Tehrani P., Golipour A., David L., Sung H.K., Beyer T.A., Datti A., Woltjen K., Nagy A., Wrana J.L. (2010). Functional genomics reveals a BMP-driven mesenchymal-to-epithelial transition in the initiation of somatic cell reprogramming. Cell Stem Cell.

[6] Hoffding M.K., Hyttel P. (2015). Ultrastructural visualization of the mesenchymal-to-epithelial transition during reprogramming of human fibroblasts to induced pluripotent stem cells. Stem Cell Res..

[7] Chronis C., Fiziev P., Papp B., Butz S., Bonora G., Sabri S., Ernst J., Plath K. (2017). Cooperative binding of transcription factors orchestrates reprogramming. Cell.

[8] Soufi A., Donahue G., Zaret K.S. (2012). Facilitators and impediments of the pluripotency reprogramming factors' initial engagement with the genome. Cell.

[9] Nichols J., Zevnik B., Anastassiadis K., Niwa H., Klewe-Nebenius D., Chambers I., Scholer H., Smith A. (1998). Formation of pluripotent stem cells in the mammalian embryo depends on the POU transcription factor Oct4. Cell.

[10] Yu J., Vodyanik M.A., Smuga-Otto K., Antosiewicz-Bourget J., Frane J.L., Tian S., Nie J., Jonsdottir G.A., Ruotti V., Stewart R. (2007). Induced pluripotent stem cell lines derived from human somatic cells. Science.

[11] Papapetrou E.P., Tomishima M.J., Chambers S.M., Mica Y., Reed E., Menon J., Tabar V., Mo Q., Studer L., Sadelain M. (2009). Stoichiometric and temporal requirements of Oct4, Sox2, Klf4, and c-Myc expression for efficient human iPSC induction and differentiation. Proc. Natl. Acad. Sci. U S A.

[12] Hammachi F., Morrison G.M., Sharov A.A., Livigni A., Narayan S., Papapetrou E.P., O'Malley J., Kaji K., Ko M.S.H., Ptashne M., Brickman J.M. (2012). Transcriptional activation by Oct4 is sufficient for the maintenance and induction of pluripotency. Cell Rep..

[13] Velychko S., Adachi K., Kim K.-P., Hou Y., MacCarthy C.M., Wu G., Schöler H.R. (2019). Excluding Oct4 from Yamanaka cocktail unleashes the developmental potential of iPSCs. Cell Stem Cell.

[14] Radzisheuskaya A., Silva J.C. (2014). Do all roads lead to Oct4? the emerging concepts of induced pluripotency. Trends Cell Biol..

[15] Huntzinger E., Izaurralde E. (2011). Gene silencing by microRNAs: contributions of translational repression and mRNA decay. Nat. Rev. Genet..

[16] Krol J., Loedige I., Filipowicz W. (2010). The widespread regulation of microRNA biogenesis, function and decay. Nat. Rev. Genet..

[17] Su Z., Yang Z., Xu Y., Chen Y., Yu Q. (2015). MicroRNAs in apoptosis, autophagy and necroptosis. Oncotarget.

[18] Hwang H.W., Mendell J.T. (2006). MicroRNAs in cell proliferation, cell death, and tumorigenesis. Br. J. Cancer.

[19] Merlin S., Follenzi A. (2019). Transcriptional targeting and MicroRNA regulation of lentiviral vectors. Mol. Ther. Methods Clin. Dev..

[20] Geisler A., Fechner H. (2016). MicroRNA-regulated viral vectors for gene therapy. World J. Exp. Med..

[21] Ruiz A.J., Russell S.J. (2015). MicroRNAs and oncolytic viruses. Curr. Opin. Virol..

[22] Brown B.D., Gentner B., Cantore A., Colleoni S., Amendola M., Zingale A., Baccarini A., Lazzari G., Galli C., Naldini L. (2007). Endogenous microRNA can be broadly exploited to regulate transgene expression according to tissue, lineage and differentiation state. Nat. Biotechnol..

[23] Colin A., Faideau M., Dufour N., Auregan G., Hassig R., Andrieu T., Brouillet E., Hantraye P., Bonvento G., Deglon N. (2009). Engineered lentiviral vector targeting astrocytes in vivo. Glia.

[24] Papapetrou E.P., Kovalovsky D., Beloeil L., Sant'angelo D., Sadelain M. (2009). Harnessing endogenous miR-181a to segregate transgenic antigen receptor expression in developing versus post-thymic T cells in murine hematopoietic chimeras. J. Clin. Invest..

[25] Sachdeva R., Jönsson M.E., Nelander J., Kirkeby A., Guibentif C., Gentner B., Naldini L., Björklund A., Parmar M., Jakobsson J. (2010). Tracking differentiating neural progenitors in pluripotent cultures using microRNA-regulated lentiviral vectors. Proc. Natl. Acad. Sci. U S A.

[26] Brown B.D., Naldini L. (2009). Exploiting and antagonizing microRNA regulation for therapeutic and experimental applications. Nat. Rev. Genet..

[27] Rota P.A., Moss W.J., Takeda M., de Swart R.L., Thompson K.M., Goodson J.L. (2016). Measles. Nat. Rev. Dis. Primers.

[28] Muhlebach M.D. (2020). Measles virus in cancer therapy. Curr. Opin. Virol..

[29] Wang Q., Vossen A., Ikeda Y., Devaux P. (2019). Measles vector as a multigene delivery platform facilitating iPSC reprogramming. Gene Ther..

[30] Driscoll C.B., Tonne J.M., El Khatib M., Cattaneo R., Ikeda Y., Devaux P. (2015). Nuclear reprogramming with a non-integrating human RNA virus. Stem Cell Res. Ther..

[31] Hiramoto T., Tahara M., Liao J., Soda Y., Miura Y., Kurita R., Hamana H., Inoue K., Kohara H., Miyamoto S. (2020). Non-transmissible MV vector with segmented RNA genome establishes different types of iPSCs from hematopoietic cells. Mol. Ther..

[32] Baertsch M.A., Leber M.F., Bossow S., Singh M., Engeland C.E., Albert J., Grossardt C., Jäger D., von Kalle C., Ungerechts G. (2014). MicroRNA-mediated multi-tissue detargeting of oncolytic measles virus. Cancer Gene Ther..

[33] Leber M.F., Baertsch M.A., Anker S.C., Henkel L., Singh H.M., Bossow S., Engeland C.E., Barkley R., Hoyler B., Albert J. (2018). Enhanced control of oncolytic measles virus using microRNA target sites. Mol. Ther. Oncolytics.

[34] Brunel J., Chopy D., Dosnon M., Bloyet L.M., Devaux P., Urzua E., Cattaneo R., Longhi S., Gerlier D. (2014). Sequence of events in measles virus replication: role of phosphoprotein-nucleocapsid interactions. J. Virol..

[35] Nathan G., Kredo-Russo S., Geiger T., Lenz A., Kaspi H., Hornstein E., Efrat S. (2015). MiR-375 promotes redifferentiation of adult human beta cells expanded in vitro. PLoS One.

[36] Hong S., Noh H., Teng Y., Shao J., Rehmani H., Ding H.F., Dong Z., Su S.B., Shi H., Kim J., Huang S. (2014). SHOX2 is a direct miR-375 target and a novel epithelial-to-mesenchymal transition inducer in breast cancer cells. Neoplasia.

[37] Selth L.A., Das R., Townley S.L., Coutinho I., Hanson A.R., Centenera M.M., Stylianou N., Sweeney K., Soekmadji C., Jovanovic L. (2017). A ZEB1-miR-375-YAP1 pathway regulates epithelial plasticity in prostate cancer. Oncogene.

[38] Soncin F., Ward C.M. (2011). The function of e-cadherin in stem cell pluripotency and self-renewal. Genes (Basel).

[39] Nethercott H.E., Brick D.J., Schwartz P.H. (2011). Derivation of induced pluripotent stem cells by lentiviral transduction. Methods Mol. Biol..

[40] Somers A., Jean J.C., Sommer C.A., Omari A., Ford C.C., Mills J.A., Ying L., Sommer A.G., Jean J.M., Smith B.W. (2010). Generation of transgene-free lung disease-specific human induced pluripotent stem cells using a single excisable lentiviral stem cell cassette. Stem Cells.

[41] Rodriguez-Madoz J.R., San Jose-Eneriz E., Rabal O., Zapata-Linares N., Miranda E., Rodriguez S., Porciuncula A., Vilas-Zornoza A., Garate L., Segura V. (2017). Reversible dual inhibitor against G9a and DNMT1 improves human iPSC derivation enhancing MET and facilitating transcription factor engagement to the genome. PLoS One.

[42] Naaman H., Rabinski T., Yizhak A., Mizrahi S., Avni Y.S., Taube R., Rager B., Weinstein Y., Rall G., Gopas J., Ofir R. (2018). Measles virus persistent infection of human induced pluripotent stem cells. Cell Reprogram..

[43] Huang H.-P., Chen P.-H., Yu C.-Y., Chuang C.-Y., Stone L., Hsiao W.-C., Li C.-L., Tsai S.-C., Chen K.-Y., Chen H.-F. (2011). Epithelial cell adhesion molecule (EpCAM) complex proteins promote transcription factor-mediated pluripotency reprogramming. J. Biol. Chem..

[44] Johannessen M., Møller S., Hansen T., Moens U., Van Ghelue M. (2006). The multifunctional roles of the four-and-a-half-LIM only protein FHL2. Cell Mol. Life Sci..

[45] Yu T., Ma Y., Wang H. (2017). EpCAM intracellular domain promotes porcine cell reprogramming by upregulation of pluripotent gene expression via beta-catenin signaling. Sci. Rep..

[46] Carpenter G., Red Brewer M. (2009). EpCAM: another surface-to-nucleus missile. Cancer Cell.

[47] Ladwein M., Pape U.F., Schmidt D.S., Schnölzer M., Fiedler S., Langbein L., Franke W.W., Moldenhauer G., Zöller M. (2005). The cell-cell adhesion molecule EpCAM interacts directly with the tight junction protein claudin-7. Exp. Cell Res..

[48] Wang Y., Baskerville S., Shenoy A., Babiarz J.E., Baehner L., Blelloch R. (2008). Embryonic stem cell-specific microRNAs regulate the G1-S transition and promote rapid proliferation. Nat. Genet..

[49] Lamb R.A., Parks G.D., Fields B.N., Howley P.M. (2007). Fields Virology.

[50] Bloyet L.M., Brunel J., Dosnon M., Hamon V., Erales J., Gruet A., Lazert C., Bignon C., Roche P., Longhi S., Gerlier D. (2016). Modulation of Re-initiation of measles virus transcription at intergenic regions by PXD to NTAIL binding strength. PLoS Pathog..

[51] Cox R.M., Krumm S.A., Thakkar V.D., Sohn M., Plemper R.K. (2017). The structurally disordered paramyxovirus nucleocapsid protein tail domain is a regulator of the mRNA transcription gradient. Sci. Adv..

[52] Teshigawara R., Hirano K., Nagata S., Ainscough J., Tada T. (2016). OCT4 activity during conversion of human intermediately reprogrammed stem cells to iPSCs through mesenchymal-epithelial transition. Development.

[53] Nishimura K., Kato T., Chen C., Oinam L., Shiomitsu E., Ayakawa D., Ohtaka M., Fukuda A., Nakanishi M., Hisatake K. (2014). Manipulation of KLF4 expression generates iPSCs paused at successive stages of reprogramming. Stem Cell Rep..

[54] Dos Santos R.L., Tosti L., Radzisheuskaya A., Caballero I.M., Kaji K., Hendrich B., Silva J.C.R. (2014). MBD3/NuRD facilitates induction of pluripotency in a context-dependent manner. Cell Stem Cell.

[55] Mikkelsen T.S., Hanna J., Zhang X., Ku M., Wernig M., Schorderet P., Bernstein B.E., Jaenisch R., Lander E.S., Meissner A. (2008). Dissecting direct reprogramming through integrative genomic analysis. Nature.

[56] Sridharan R., Tchieu J., Mason M.J., Yachechko R., Kuoy E., Horvath S., Zhou Q., Plath K. (2009). Role of the murine reprogramming factors in the induction of pluripotency. Cell.

[57] Jaenisch R., Jahner D., Nobis P., Simon I., Lohler J., Harbers K., Grotkopp D. (1981). Chromosomal position and activation of retroviral genomes inserted into the germ line of mice. Cell.

[58] Ebrahimi B. (2015). Reprogramming barriers and enhancers: strategies to enhance the efficiency and kinetics of induced pluripotency. Cell Regen..

[59] Golipour A., David L., Liu Y., Jayakumaran G., Hirsch C.L., Trcka D., Wrana J.L. (2012). A late transition in somatic cell reprogramming requires regulators distinct from the pluripotency network. Cell Stem Cell.

[60] Okita K., Ichisaka T., Yamanaka S. (2007). Generation of germline-competent induced pluripotent stem cells. Nature.

[61] Subramanyam D., Lamouille S., Judson R.L., Liu J.Y., Bucay N., Derynck R., Blelloch R. (2011). Multiple targets of miR-302 and miR-372 promote reprogramming of human fibroblasts to induced pluripotent stem cells. Nat. Biotechnol..

[62] Gandra U.R., Sinopoli A., Moncho S., NandaKumar M., Ninkovic D.B., Zaric S.D., Sohail M., Al-Meer S., Brothers E.N., Mazloum N.A. (2019). Green light-responsive CO-releasing polymeric materials derived from ring-opening metathesis polymerization. ACS Appl. Mater. Interfaces.

[63] Lei Z., Maeda T., Tamura A., Nakamura T., Yamazaki Y., Shiratori H., Yashiro K., Tsukita S., Hamada H. (2012). EpCAM contributes to formation of functional tight junction in the intestinal epithelium by recruiting claudin proteins. Dev. Biol..

[64] Kärber G. (1931). Beitrag zur kollektiven Behandlung pharmakologischer Reihenversuche. Arch. Exp. Pathol. Pharmakol..

