# Supplementary material for: miRNA-mediated control of exogenous OCT4 during mesenchymal-epithelial transition increases measles vector reprogramming efficiency
Source: Mol Ther Methods Clin Dev. 2021 Nov 29;24:48–61. doi: 10.1016/j.omtm.2021.11.012 (PMC8683617; doi:10.1016/j.omtm.2021.11.012)
Supplement: Document S1. Figures S1–S6 and Tables S1–S3 [file mmc1.pdf]

OMTM, Volume *24*

Supplemental information

miRNA-mediated control of exogenous *OCT4* during mesenchymal-epithelial transition increases measles vector reprogramming efficiency

Ramya Rallabandi, Brenna Sharp, Conrad Cruz, Qi Wang, Alexis Locsin, Christopher B. Driscoll, Ella Lee, Tim Nelson, and Patricia Devaux

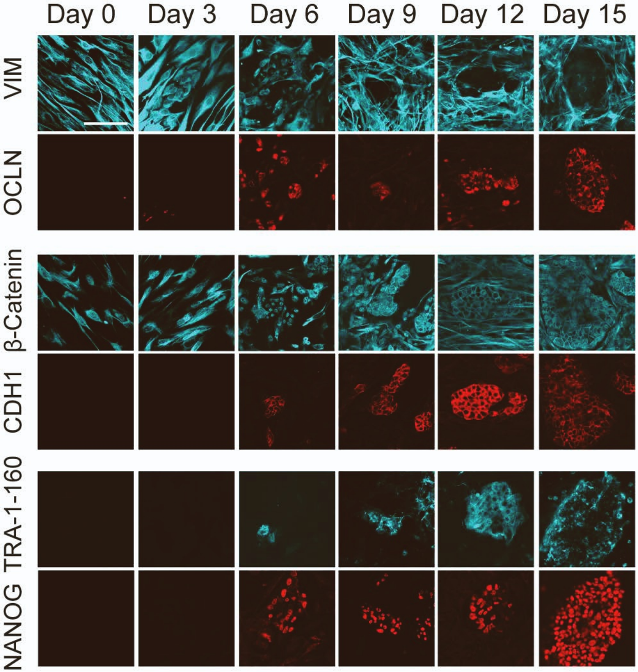

Figure S1: Human fibroblasts start MET at day 6 for LV reprogramming, Related to Figure 2
A) Double immunofluorescent labeling of specified markers at Day 0, 3, 6, 9, 12 and 15 of NHF reprogramming. The Scale bars is 100 μm.

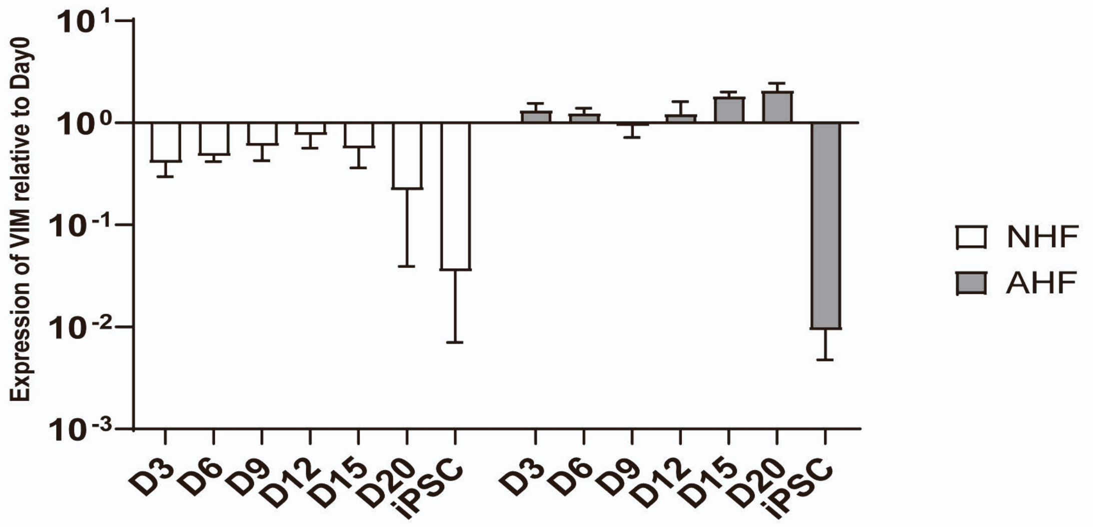

Figure S2. The relative expression of Vimentin (VIM) during MeV reprogramming, Related to Figure 2
qPCR analysis of mesenchymal marker, VIM during day 0, 3, 6, 9, 12, 15 and iPSC of NHF (white bars) and AHF (grey bar) reprogramming using MV(O)(SK)(M). All values are relative to day 0 and normalized to GAPDH. Error bars indicate mean ± SD from 3 independent experiments.

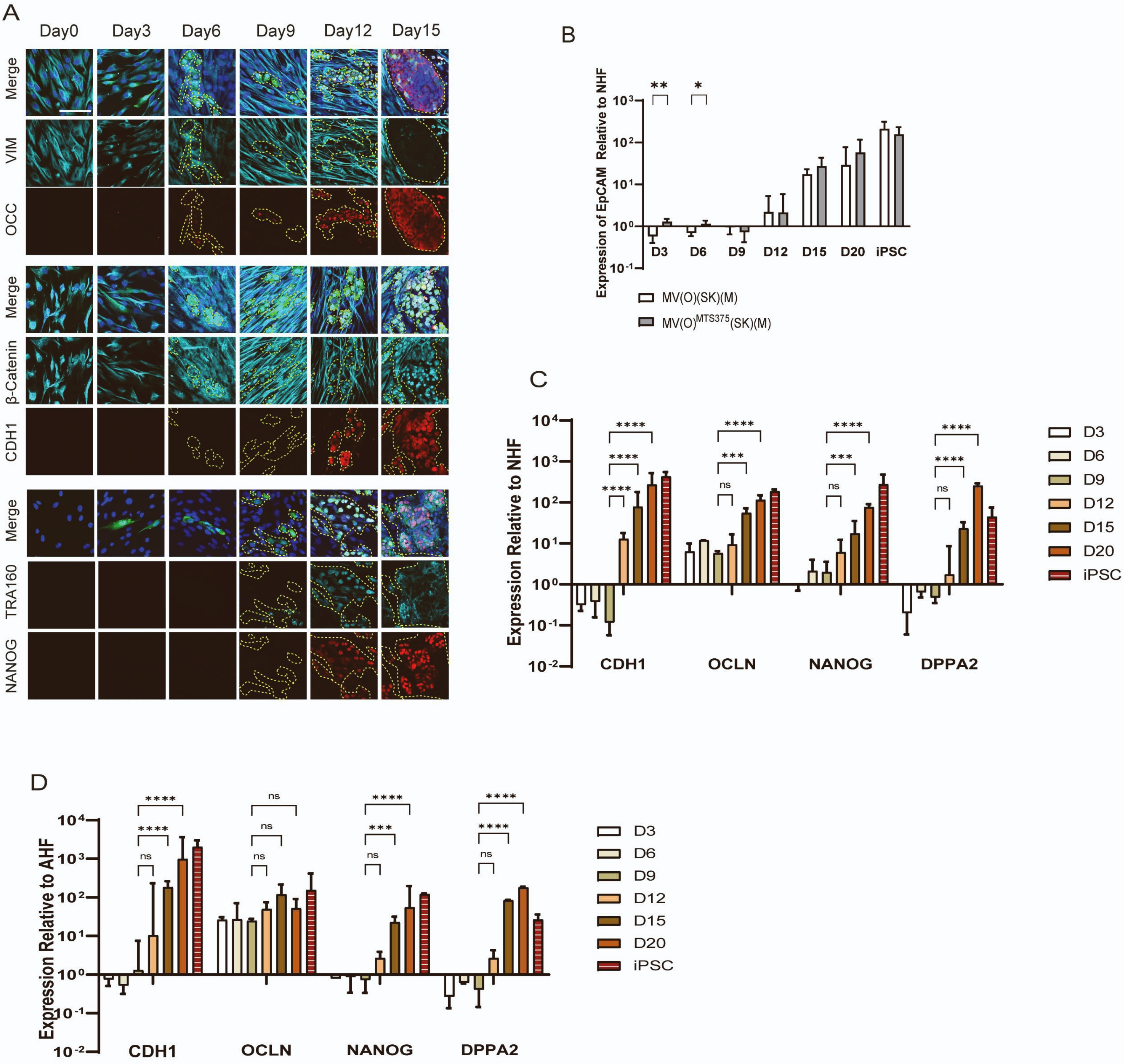

Figure S3. Characterization of MET for MV(O)^{MTS375}(SK)(M) reprogramming on NHF. Related to Figure 3

(A) Double immunofluorescent labeling of specified markers at Day 0, 3, 6, 9, 12 and 15 of NHF reprogramming using MV(O)^{MTS375}(SK)(M). Merge is presented as overlays of indicated antibody staining, GFP and Dapi staining. GFP+ cells = Yellow dashed lines. The Scale bars = 100 μm.

(B) qPCR analysis of relative expression of endo. EPCAM during NHF reprogramming with indicated vectors. All values are relative to day 0 and normalized to GAPDH (**: $P < 0.01$, ***: $P < 0.001$, ****: $P < 0.0001$). Error bars indicate mean ± SD from 3 independent experiments.

(C-D) qRT-PCR analysis of endo markers (indicated) in MV(O)^{MTS375}(SK)(M) reprogramming on NHF (C) OR AHF (D). All values are relative to day 0 and normalized to GAPDH. Error bars indicate mean ± SD from 3 independent experiments.

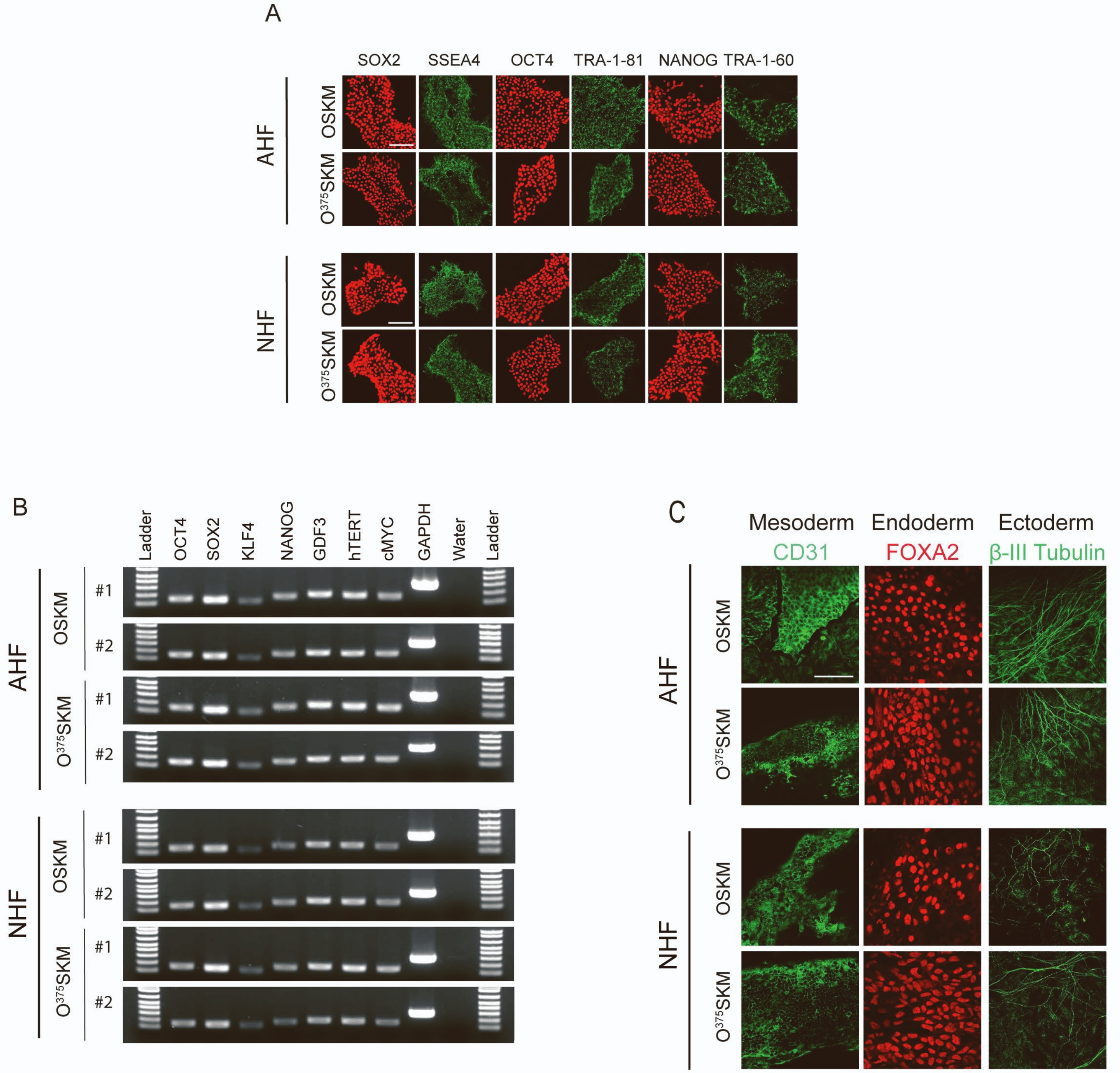

Figure S4: Characterization of MV(O)(SK)(M) and MV(O)^{MTS375}(SK)(M) derived iPSC clones, Related to Figure 2 and Figure 3 (A) Representative images of immunofluroscent labelled iPSC clones expressing the pluripotency markers, derived from AHF(top) and NHF(bottom) reprogramming using specified vectors, MV(O)(SK)(M)-(OSKM) and MV(O)^{MTS375}(SK)(M)-(O^{MTS375}SKM). Scale bar=100 µm. (B) RT-PCR analysis of iPSC clones, derived from MV(O)(SK)(M) and MV(O)^{MTS375}(SK)(M) reprogramming on both AHFs and NHFs, expressing endogenous iPSC markers. (C) Spontaneous differentiation of iPSC clones from specified vectors to mesoderm (CD31), endoderm (FOXA2) and ectoderm (β-III tubulin) lineages. Scale bars = 100 µm.

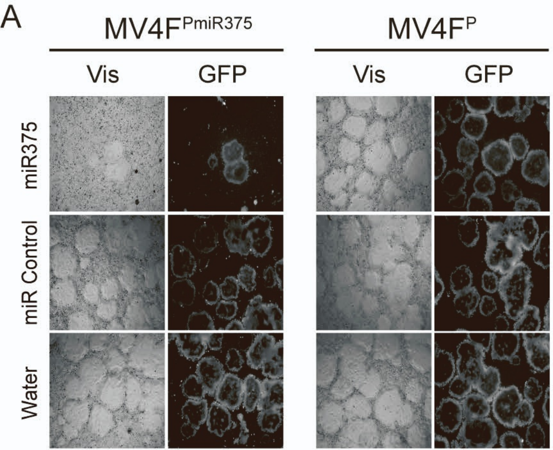

Figure S5. MV4FP^{MTS375} vector fails to propagate in presence of miR375, Related to Figure 5.
Representatitive bright field and fluorescent (GFP) images of vector propagation of indicated vectors in 293LVH cells transfected with miR-375, scarmbled miRNA or water.

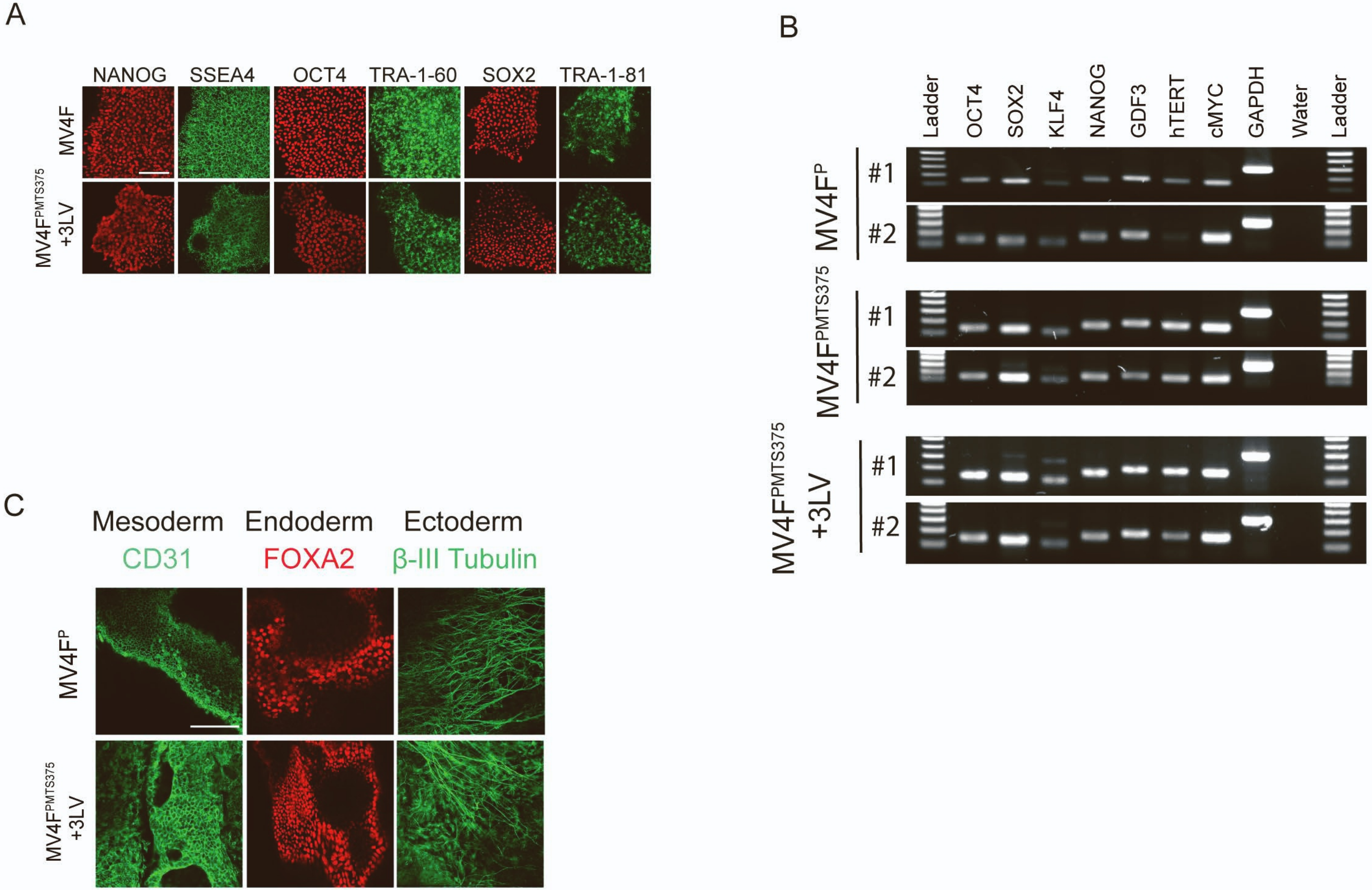

Figure S6. Characterization of $MV4F^{P}$ and $MV4F^{PMTS375}$ derived iPSC clones. Related to Figure 5.
(A) Representative confocal images of iPSC clones expressing the pluripotency markers, derived from NHF reprogramming using specified vectors. Scale bar = 100 μm. (B) RT-PCR analysis of iPSC clones, derived from NHF reprogramming using specified vectors, expressing endogenous iPSC markers. (C) Spontaneous differentiation of iPSC clones from indicated vectors to mesoderm (CD31), endoderm (FOXA2) and ectoderm (β-III tubulin) lineages. Scale bar = 100 μm

Supplemental Information

Table S1: Antibodies for Immunofluorescence

**PRIMARY ANTIBODIES**	**COMPANY**	**CAT**
E-cadherin Rabbit Anti-human	Cell Signaling Technology Inc	3195S
Occludin Mouse Anti-human	Invitrogen	711500
Vimentin Rat Anti-human	R&D	MAB2105
β-catenin Mouse Anti-human	R&D	MAB2081
NANOG Rabbit Anti-human	Abcam	AB21624
TRA-1-60 Mouse Anti-human	Stemgent	09-0010
OCT4 Rabbit Anti-human	Cell Signaling Technology Inc	2750S
SOX2 Rabbit Anti-human	Cell Signaling Technology Inc	2748S
KLF4 Mouse Anti-human	Stemgent	09-0021
c-MYC Mouse Anti-human	Santa Cruz Biotechnology	sc-40
**SECONDARY ANTIBODIES**	**COMPANY**	**CAT**
Alexa Fluor 647 Donkey Anti-Mouse	Life Technologies	A31571
Alexa Fluor 594 Donkey Anti-Rabbit	Life Technologies	A21207
Alexa Fluor 488 Donkey Anti-Mouse	Life Technologies	A21202
Alexa Fluor 647 Goat Anti-Rat	Life Technologies	A21247

Table S2: Antibodies for Western Blot

**PRIMARY ANTIBODIES**	**COMPANY**	**CAT**
OCT4 Rabbit Anti-human	Cell Signaling Technology Inc	2750S
SOX2 Rabbit Anti-human	Cell Signaling Technology Inc	2748S
c-MYC Mouse Anti-human	Santa Cruz Biotechnology	sc-40
β-actin peroxidase-conjugated	Sigma-Aldrich	A3854
Mouse anti- Measles N	Cl25 [31]	
rabbit anti-P254	P254[66]	
**SECONDARY ANTIBODIES**	**COMPANY**	**CAT**
Peroxidase-conjugated Anti-mouse	Calbiochem,	401215
Peroxidase-conjugated anti-rabbit	Jackson ImmunoResearch	111-035-003

Table S3: List of primers and probes used for gene expression analyses

TARGET	TYPE	SEQUENCE
CDH1	FWD PRIMER	GAACAGCACGTACACAGCCCT
	REV PRIMER	GCAGAAGTGTCCCTGTTCCAG
	PROBE	ATCATAGCTACAGACAATGGTTCTCCA
CLDN7	FWD PRIMER	CGCCTTGGTAGCTTGCTCCT
	REV PRIMER	AGCAGTGCACCTCCCAGGAT
	PROBE	AG GGC AGA CCC TGC CCA GCC
c-MYC	FWD PRIMER	CGGTGCAGCCGTATTTCTAC
	REV PRIMER	GGCAGCAGCTCGAATTTCTT
	PROBE	CCCGGCGCCCAGCGAGGATA
DPPA2	FWD PRIMER	GTTGGCATCATGGGCAAGAA
	REV PRIMER	TTGTGTCTGCCGAGAGAAGT
	PROBE	ACCACCTGACGCCAGAGGCTTGC
ENDO *OCT4*	FWD PRIMER	GCCGGGCTGGGTTGATCCTC
	REV PRIMER	CCCACACCTCAGAGCCTGGC
	PROBE	CCCGGCCCGATTCCTGGCCCTC
EPCAM	FWD PRIMER	GGCTCTTTAAGGCCAAGCAG
	REV PRIMER	CCAGTAGGTTCTCACTCGCT
	PROBE	ACGGCACCTCCATGTGCTGGT
GAPDH	FWD PRIMER	ACCCAGAAGACTGTGGATG
	REV PRIMER	TCAGCTCAGGGATGACCTT
	PROBE	CCCACAGCCTTGGCAGCGC
EXO *KLF4*	FWD PRIMER	GCTGGAAGTTCGCTAGAAGC
	REV PRIMER	CATGTGGAGAGCCAGGTGAT
	PROBE	TGCGACAGGGCCTTCTCTAGATCCG
Measles N	FWD PRIMER	GGCCCAGCAGAGCAAGTGAT
	REV PRIMER	TTGGCTGGACTCCGTTGCAG
	PROBE	AGCTGCCCATCTTCCAACCGGCA
NANOG	FWD PRIMER	CCTCCAGCAGATGCAAGAAC
	REV PRIMER	TCCCTGGTGGTAGGAAGAGT
	PROBE	ACGCAGAAGGCCTCAGCACCT
OCLN	FWD PRIMER	TAACTTCGCCTGTGGATGAC
	REV PRIMER	CTCTTTGACCTTCCTGCTCTT
	PROBE	CAGCCTCGTTACAGCAGCGGT
EXO *OCT4*	FWD PRIMER	TGCAAAGCAGAAACACTCGT
	REV PRIMER	GGCTTAGGGCATTGCAGAAA
	PROBE	TGCCTCTGACCCTGTTCTC
VIM	FWD PRIMER	GGATGTTGACAATGCGTCTCT
	REV PRIMER	GGACATGCTGTTCCTGAATCTGAG
	PROBE	TTTGCGTTCAAGGTCAAGACGTGC

microRNA Probe+Primers for Taqman qRT-PCR

microRNA	Commercial Code
miR375	Thermofisher- 4427975 Assay ID 000564
RNU6B	Thermofisher- 4427975 Assay ID 001093
miR372-3p	Thermofisher- 4427975 Assay ID 000560
miR373-3p	Thermofisher- 4427975 Assay ID 000561
miR302a-3p	Thermofisher- 4427975 Assay ID 000529

All sequences are human and written in the 5' to 3' direction with probe of 5'FAM and 3'TAMRA
